# Effects of Electrodeposition Mode and Deposition Cycle on the Electrochemical Performance of MnO_2_-NiO Composite Electrodes for High-Energy-Density Supercapacitors

**DOI:** 10.1371/journal.pone.0154566

**Published:** 2016-05-16

**Authors:** Rusi, S. R. Majid

**Affiliations:** Centre for Ionics University of Malaya, Faculty of Science, University of Malaya, 50603 Kuala Lumpur, Malaysia; The University of Akron, UNITED STATES

## Abstract

Nanostructured network-like MnO_2_-NiO composite electrodes were electrodeposited onto stainless steel substrates via different electrodeposition modes, such as chronopotentiometry, chronoamperometry, and cyclic voltammetry, and then subjected to heat treatment at 300°C for metal oxide conversion. X-ray diffraction, field emission scanning electron microscopy, and transmission electron microscopy were used to study the crystalline natures and morphologies of the deposited films. The electrochemical properties were investigated using cyclic voltammetry and charge/discharge tests. The results revealed that the electrochemical performance of the as-obtained composite electrodes depended on the electrodeposition mode. The electrochemical properties of MnO_2_-NiO composite electrodes prepared using cyclic voltammetry exhibited the highest capacitance values and were most influenced by the deposition cycle number. The optimum specific capacitance was 3509 Fg^−1^ with energy and power densities of 1322 Wh kg^−1^ and 110.5 kW kg^−1^, respectively, at a current density of 20 Ag^−1^ in a mixed KOH/K_3_Fe(CN)_6_ electrolyte.

## Introduction

Interest in supercapacitors has increased recently because of the high demand for energy production and storage resulting from rapid population growth and high energy consumption in the industrial sector [1]. This area of research not only faces the challenge of maintaining renewable and sustainable energy sources but must also find a method to store this energy effectively [2–3]. In general, such energy can be stored in three ways: electrical, chemical, and electrochemical energy storage. In chemical storage, the energy takes the form of potential energy that can be released by chemical reactions. When chemical energy is converted into electricity, this method is described as electrochemical energy storage and is commonly used in rechargeable batteries. Finally, in electrical storage, an electric field is used to store the energy, as in electrochemical capacitors (ECs). Electrical energy-storage devices have received substantial attention because of their advantages, such as high power density and long cycle life, compared to batteries [2–5].

The energy in ECs can be stored via two mechanisms: electrical double-layer capacitance (EDLC) and pseudocapacitance. The type of mechanism depends on the type of electrode material [5–6]. Several alternative electrode materials have been studied, including carbonaceous materials, conducting polymers, metal hydroxides, and metal oxides [2,7]. Carbonaceous materials with high surface areas, such as activated carbon, graphene, and carbon nanotubes, are commonly used for EDLC, where the capacitance is confined to the accumulated charge at the electrode/electrolyte interface. The fast charge-storage mechanism of EDLC produces high-power density (~10 kW/kg) but low energy density (~5 Wh/kg) [8]. To increase ECs’ energy densities, metal oxides, such as RuO_2_, MnO_2_, NiO, Fe_3_O_4,_ and Co_3_O_4_, are used. These materials offer high specific capacitance, mainly because of their fast surface reversible redox reactions, which help to enhance the energy density [3,9]. In acidic electrolytes, the noble metal oxide RuO_2_ exhibits a high specific capacitance of ~720 Fg^−1^ because of its high electrical conductivity and pseudo-faradaic reactions. However, this material’s high cost and potential to harm the environment have limited its commercial application in supercapacitors. Therefore, base metal oxides, such as MnO_2_, NiO, and Fe_3_O_4_, have been proposed as promising electrode materials because of their environmental compatibility, low cost, abundant availability, environmental compatibility, and wide potential windows [9–10]. MnO_2_ is a potential alternative electrode material for ECs because it exhibits these favourable properties, and it has been broadly studied as a cathode material for batteries. Based on a one-electron redox reaction per Mn atom, the theoretical specific capacitance of MnO_2_ is high (1370 Fg^−1^) and can be achieved using nanometer-scale thin films and nano-sized particles [11]. Another candidate material with a high theoretical specific capacitance value is NiO (3750 Fg^−1^), which also exhibits good electrochemical and thermal stability [12]. Because of its sufficient specific surface area, porous NiO can provide a short diffusion pathway for electrolyte cations to electroactive sites on the electrode and increase the rates of faradaic redox reactions [13]. One promising approach to improve the performance of metal oxide electrodes is by combining two metal oxides in a composite and thereby enhancing ECs’ specific capacitance and energy density [14].

Binder-free electrodes are known to exhibit higher specific capacitance because of the unclogged surfaces of their active materials and good connectivity between particles. Numerous reports on binder-free electrodes have been published in the literature. Electrodeposition is advantageous because it consumes less energy, is inexpensive and simple, and can be easily implemented and used to control the resulting morphology [15–16]. Additionally, the deposited electrode can be used directly after heat treatment without any additional processing steps. Currently, three electrodeposition modes that can be employed to deposit metal oxide: potentiodynamic (cyclic voltammetry [CV]), potentiostatic (chronoamperometry [CA]), and galvanostatic (chronopotentiometry [CP]). These different electrodeposition modes significantly affect the resulting films’ surface morphologies, crystal structures, and performance in corresponding applications [16–17]. Dubal et al. reported comparative studies on the electrochemical performance of deposited electrodes. In these studies, Dubal et al. claimed that the potentiodynamic electrodeposition mode is the most suitable method for depositing MnO_2_ to maximize its specific capacitance (237 Fg^−1^). The structures resulting from different electrodeposition modes have also been investigated [17]. Similar conclusions were reached based on the work described by Sorkhabi et al [15]. Regarding the electrode performance of potentiodynamically deposited NiO, Jagadale and his co-workers [15] showed that the nanoflake-like morphology of a NiO thin film exhibited a maximum specific capacitance of 222 Fg^−1^.

This study investigated the effects of different electrodeposition modes on the capacitance performance of NiO-MnO_2_ composite electrodes in Na_2_SO_4_ electrolyte. To the best of our knowledge, no comparative study of composite binary metal oxides prepared by different electrodeposition modes has been reported previously, except for one report on single-metal oxide electrodes. We show that all the studied MnO_2_-NiO samples contained homogeneous distributed porous structures. CV measurements revealed that potentiodynamically deposited MnO_2_-NiO electrodes had high specific capacitance, which can be attributed to the presence of pseudocapacitive MnO_2_ nano-particles connected to the electrically conductive NiO. Finally, we demonstrated that the morphological structure, deposit thickness, and electrochemical performance were influenced by the number of deposition cycles. The capacitance decreased as the number of deposition cycles increased because of the ineffective wettability properties at the electrode/electrolyte interfaces and the lower intercalation/deintercalation activity into or from the electrodes. The optimum electrode was studied in three different electrolytes, i.e., Na_2_SO_4_, KOH, and mixed KOH/ K_3_Fe(CN)_6_. We previously addressed MnO_2_-NiO electrodes deposited via CP with different Ni concentrations in the deposition solution; please consult that work for the experimental details [14].

## Materials and Methods

All the chemicals used in this study were analytical grade and were used without further purification. The deposition electrolyte was prepared by mixing 0.01-M Mn acetate tetrahydrate (Mn(CH_3_COO)_2_.4H_2_O), 0.25-M Ni acetate tetrahydrate (Ni(CH_3_COO)_2_.4H_2_O), and 0.8-M sulfuric acid (H_2_SO_4_) to obtain a homogeneous solution. The Mn-Ni oxide (MnO_2_-NiO) films were deposited onto 2×2-cm^2^ stainless steel (SS) substrates using different deposition modes. The deposition process was conducted in three electrode systems with a carbon rod, Ag/AgCl, and SS as the counter, reference, and working electrodes, respectively. The three electrodeposition modes studied here were CP, CA, and CV. To deposit the Mn-Ni hydroxide using the CP mode, the current was maintained at 8 mA for 10 min of deposition time (sample named CP), whereas a constant voltage of 1.5 V was applied to deposit the Mn-Ni hydroxide via CA (sample named CA). In the CV mode, the electrodeposition was conducted in the voltage range from 0 to 1 V at a scan rate of 20 mVs^−1^ with 7 deposition cycle (sample named CY7). Finally, the deposited samples were rinsed with distilled water before being subjected to post-heating at 300°C for 6 h. The CV mode (CY7) generated the best results, and thus, the effect of the number of deposition cycles in CV mode was studied further. To this end, the numbers of deposition cycles tested were 4 cycles, 10 cycles, and 13 cycles, and the corresponding electrodes were named CY4, CY10, and CY13, respectively.

The powder X-ray diffraction (XRD) patterns were recorded using a D8 Advance X-Ray diffractometer-Bruker AXS instrument with CuK_α_ monochromatic radiation at 40 kV. The elemental composition was studied using energy-dispersive X-ray (EDX) spectroscopy (Oxford Instruments). Field emission scanning electron microscopy (FESEM) and transmission electron microscopy (TEM) images of the electrodes were captured using Jeol JSM-7600F and Jeol JEM 2100F instruments. The amount of material loaded onto the SS substrate was weighed using a microbalance (Sartorius CPA225D). The electrochemical measurements were performed using three-electrode methods and an Autolab PGSTAT30 potentiostat/galvanostat. Platinum wire was used as a counter electrode, and Ag/AgCl was used as the reference electrode. CVs were recorded in a potential window ranging from −1 to +1 V versus Ag/AgCl with a scan rate of 5 mVs^−1^, whereas galvanostatic cycling was recorded in a potential window of −0.6 V to 1 V with a current density of 1 Ag^−1^. Electrochemical impedance spectroscopy (EIS) was conducted in the frequency range of 10 mHz to 100 kHz. The specific capacitance (C) can be calculated from both the CV and charge/discharge (CD) results using Eqs 1 and 2, respectively [18]:

C=(∫Idt)/(ΔV×m) (1)

where *I* is the oxidation/reduction current, *dt* is the time differential, *m* is the mass of the active material, and *ΔV* is the operating potential.

C=I/((dV/dt)×m) (2)

where *I* is the discharge current, $\frac{dV}{dt}$ is the change of the discharge potential with the discharge time, and *m* is the mass of the active materials.

Additionally, the energy (E) and power (P) densities can be expressed as Eqs 3 and 4, respectively [19]:

$$E=(CV^{2})/2$$ (3)

$$P=V^{2}/(4R_{s})$$ (4)

where *V* is the operating potential, and *R*_*s*_ is the equivalent inner resistance of the device.

## Results and Discussion

**Fig 1:**
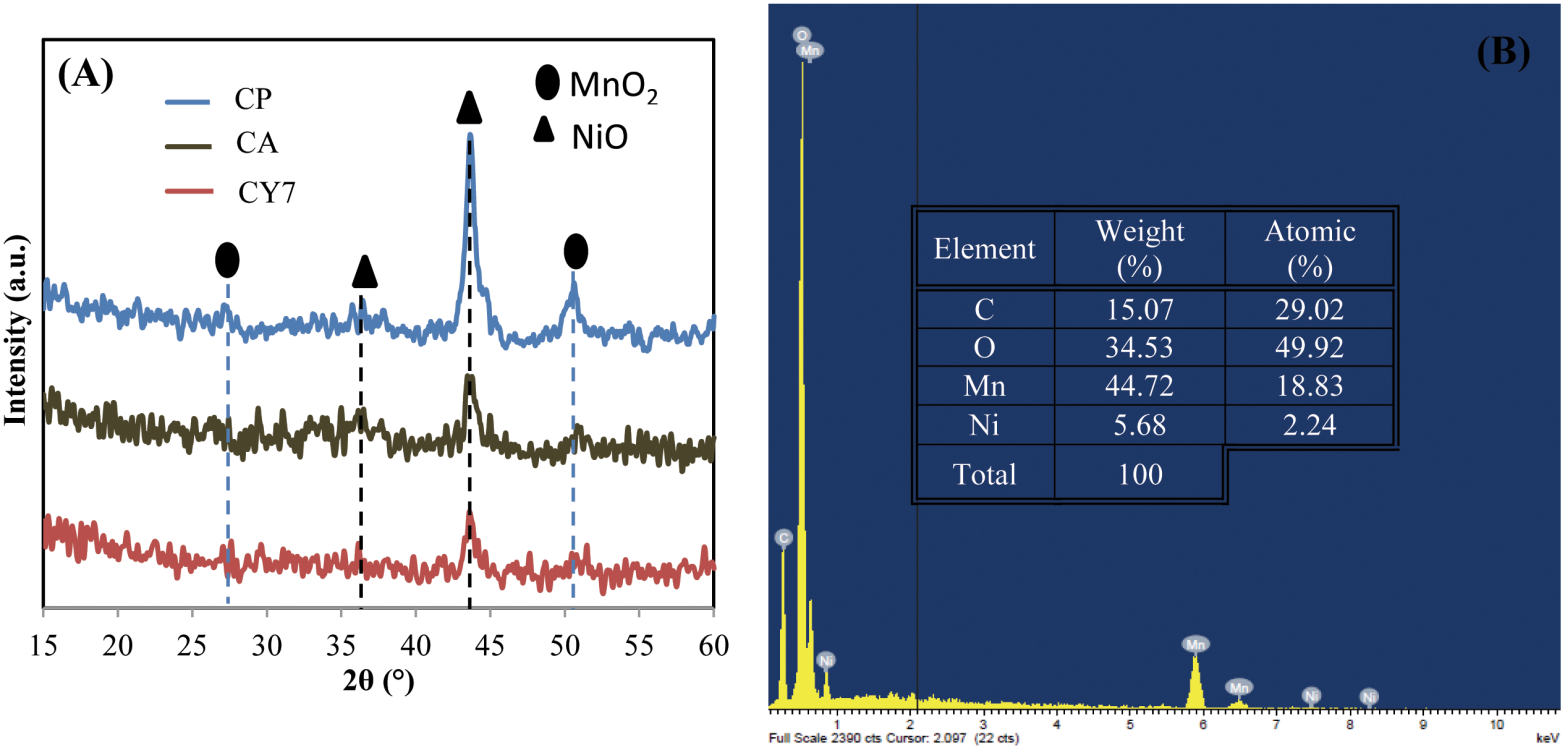
(A) XRD patterns of the products prepared using different electrodeposition modes. (B) EDX of the CY7 electrode.

The crystal structure of Mn-Ni oxide was examined by XRD. The XRD patterns of Mn-Ni oxide powder deposited by CP, CA, and CY7 are shown in Fig 1A. All the XRD peaks of the as-obtained samples can be indexed to α-MnO_2_ [JCPDS card no. 44–0141] and NiO [JCPDS card no. 04–0835]. The intensities of the MnO_2_ and NiO peaks confirm that the samples’ crystalline natures are influenced by the deposition mode, which can change their crystal sizes and amorphousness. The patterns revealed the formation of MnO_2_-NiO with pronounced diffraction peaks at angles (2θ) of approximately 27.2° and 50.5°, which correspond to the (310) and (411) planes of α-MnO_2_, and 2θ = 36.3° and 43.7°, which correspond to the (111) and (200) diffraction planes of NiO [4,20–21]. The synthesized CY7 electrode had relatively weak peak intensities, suggesting that the deposited MnO_2_-NiO particles are in a poorly crystalline state, which could contribute to these particles having a larger specific area than those in a highly crystalline film [21]. No change in the chemical compositions of MnO_2_ and NiO was noted between the electrodes deposited using different modes. The EDX results of the deposited MnO_2_-NiO powder is shown in Fig 1B. The compositions and weights (%) of Ni, Mn, and O are displayed and confirmed the successful deposition of MnO_2_-NiO on the SS substrates.

The possible mechanisms underlying the deposition binary metal oxides via different electrodeposition modes can be described by the equations presented below. When the potential is applied to the deposition cell, H_2_O is reduced on the working electrode, as described by Eq 5.

$${\text{2H}}_{\text{2}}\text{O }+\text{ 2e }\to {\text{ H}}_{\text{2}}{\text{+2OH}}^{-}$$ (5)

Water reduction produces OH^−^ ions, which are believed to play a vital role in the deposition of metal oxides. The condensation of Mn^+^ and Ni^+^ ions occurs where the OH^−^ ions are localized. The binding affinity between Mn^+^/Ni^+^ cations and OH^−^ leads to the nucleation of metal hydroxide particles. Metal oxide particles then come to rest on the substrate during the annealing process after the hydroxide is efficiently oxidized in the complex, as described by Eqs 6 and 7 [14,22].

$${\text{Mn}}^{\text{2+}}{\text{+ OH}}^{-}\;\to \text{ MnOOH }\to {\text{ MnO}}_{\text{2}}$$ (6)

$${\text{Ni}}^{\text{2+}}{\text{+ OH}}^{-}\;\to \text{ NiOH }\to \text{ NiO}$$ (7)

The nucleation and growth of the oxide particles may be affected by the electrodeposition mode, resulting in alterations of the metal oxide particles’ morphologies, but the elemental properties are not affected, as demonstrated by XRD.

**Fig 2:**
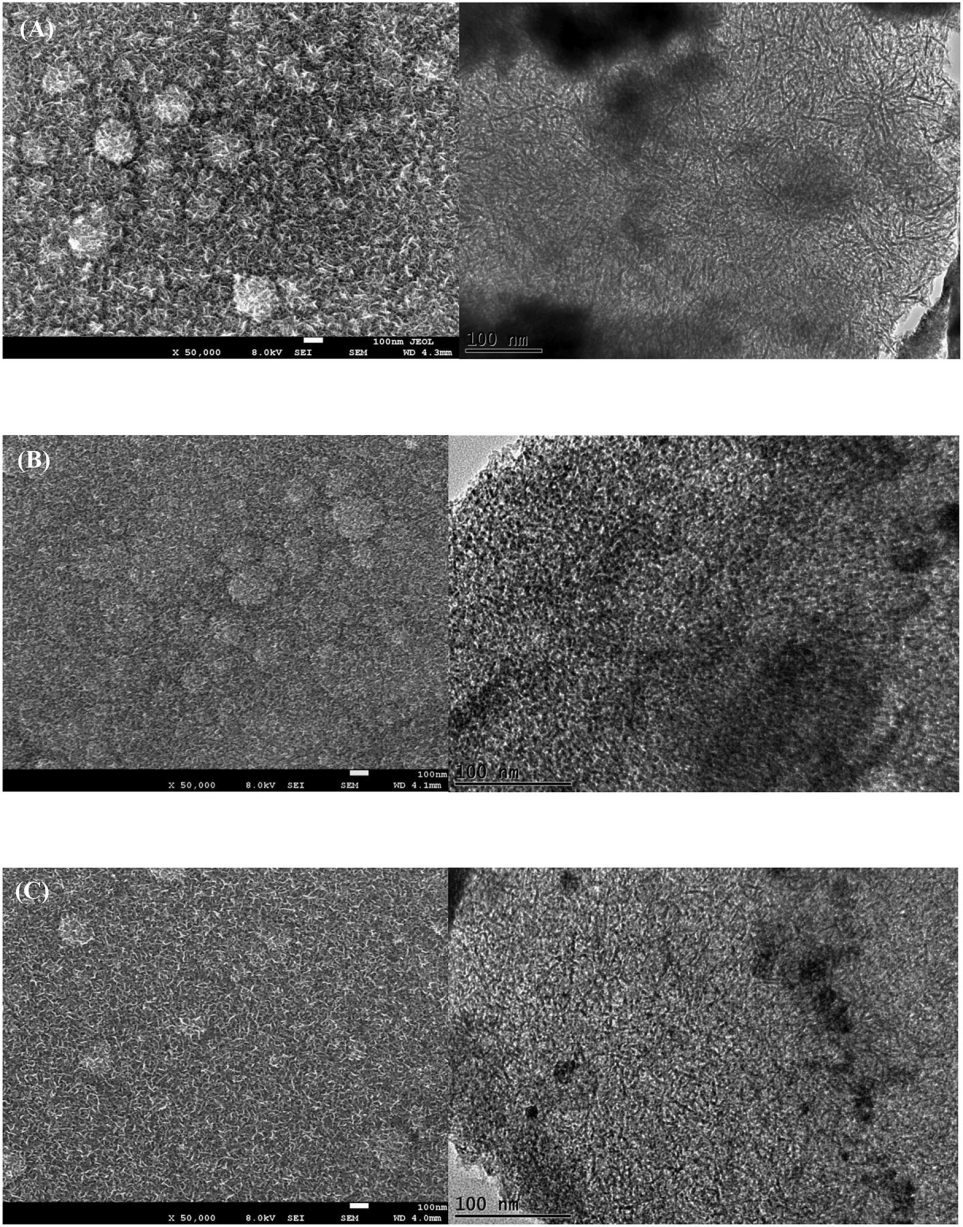
FESEM (left) and TEM (right) images of (A) CP, (B) CA, and (C) CY7 electrodes.

The as-prepared Mn-Ni oxides obtained via CA, CP, and CY7 had the same crystal structures but different morphologies, which were investigated by FESEM and TEM, as shown in Fig 2. The FESEM images revealed that all of the MnO_2_-NiO samples contained homogeneously distributed porous structures. Fewer particle agglomerations were found in the CY7 electrode sample (Fig 2C). Closure inspection of the TEM images revealed that the obtained CA and CY7 electrodes displayed smaller pore sizes (~2–4 nm) than the CP electrode (~10–15 nm). These results indicate that the CA and CY7 samples are mesoporous materials, which allow electrolyte ions to easily diffuse through the electrode bulk and, thus, enhance the number of faradic reactions that occur during electrochemical operation [9–10].

**Fig 3:**
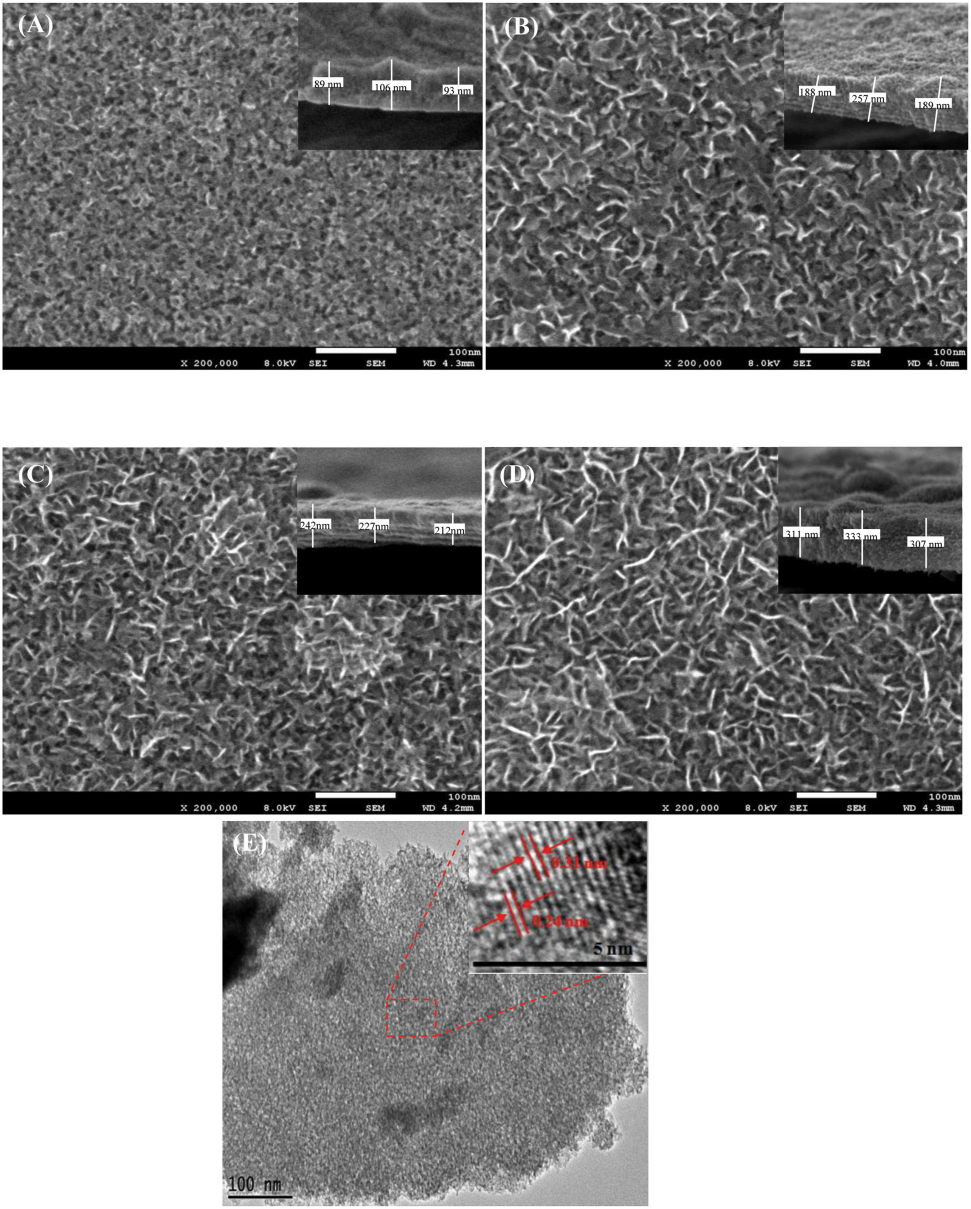
FESEM images of (A) CY4, (B) CY7, (C) CY10, and (D) CY13 and (E) TEM images of CY4 (inset: lattice spacing).

The morphologies of the electrodes produced using different numbers of deposition cycles was investigated using FESEM (Fig 3). This figure shows that varying the number of deposition cycles resulted in different morphologies. After the fewest number of deposition cycles tested (CY4 electrode, Fig 3A), clear interconnected metal oxide particles with a porous structure were observed; however, the flake structure was not able to grow because of the limited number of cycles. Fig 3B–3D) show the formation of a clear flake structure and reveal that it becomes increasingly flakes as the number of deposition cycles increases. These compact/dense flakes increase the deposited mass load. The possible mechanisms at work during the deposition process are explained below. The initial formation of stable interconnected MnO_2_-NiO is believed to occur early during the deposition (around the forth cycle) because of the instantaneous nucleation process. Subsequently, a progressive nucleation process takes place, in which the flake structure grows on the top of the previously formed nuclei. As the number of deposition cycles increases, the growth rate of the progressive nucleation also increases, resulting in denser flakes and compact grains [23–24].

The TEM image of a CY4 electrode is shown in Fig 3E. This figure reveals no flake structures in the deposits and more highly distributed pores than CY7 (Fig 2C). The elemental composition of the MnO_2_-NiO deposits in the CY4 electrode was analysed using high-resolution TEM lattice images (Fig 3E), (inset)). These images confirmed that the measured interplanar spacing corresponds to MnO_2_ and NiO. Furthermore, the periodic lattice fringe of 0.31 nm corresponds to the (310) plane of crystalline α-MnO_2_, whereas the lattice spacing of 0.24 nm corresponds to the (111) plane of NiO. These interplanar spacings are in agreement with the XRD results of MnO_2_-NiO [25–26].

**Fig 4:**
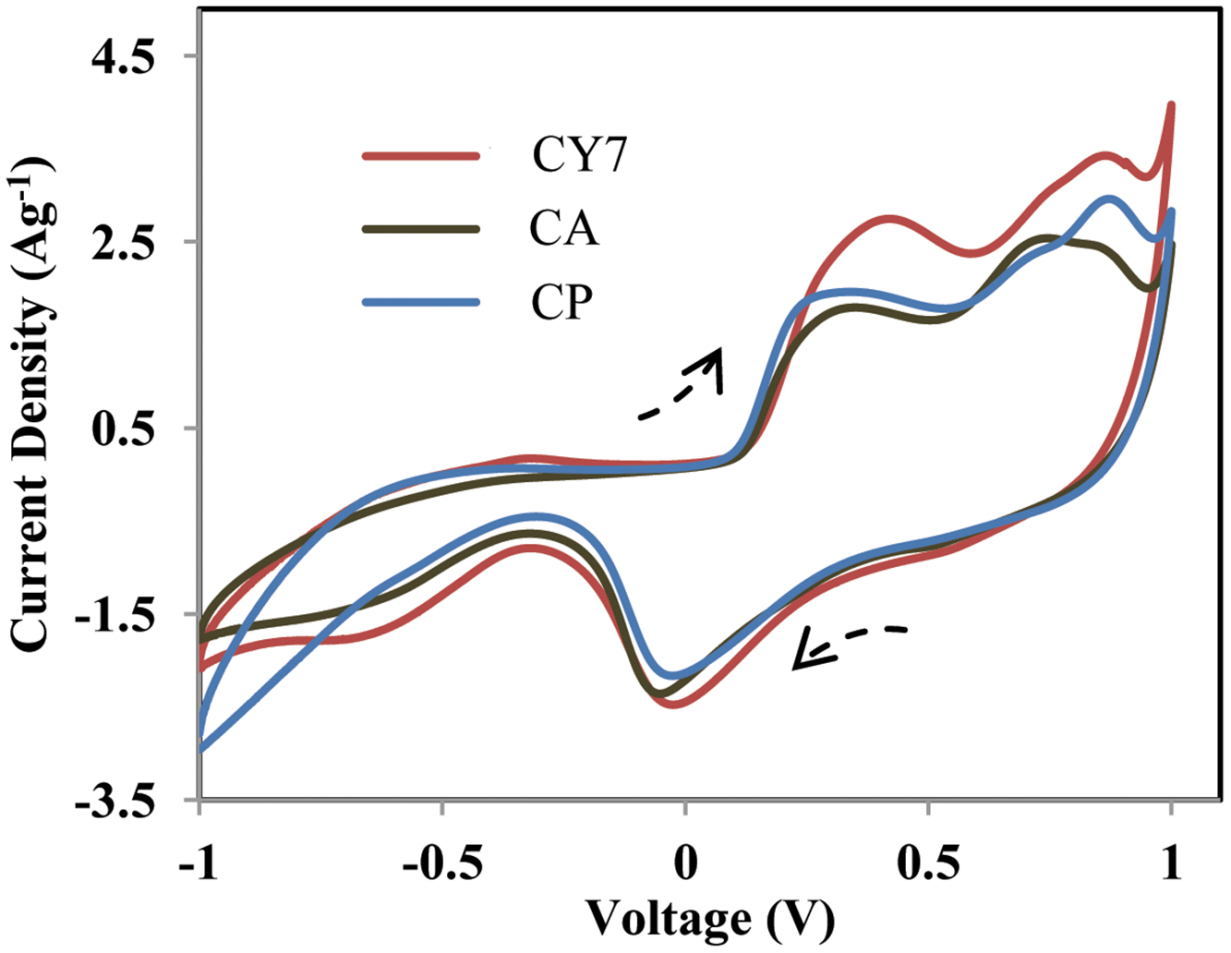
CV profiles of as-prepared electrodes obtained using different electrodeposition modes.

To determine the supercapacitive behaviours of the studied electrodes, CV was performed in 0.5-M Na_2_SO_4_ in the potential window from −1 to 1 V at a scan rate of 1 mVs^−1^ and are displayed in Fig 4. A pair of symmetric anodic and cathodic peaks are clearly evident in each CV profile, implying the existence of two reversible faradic reactions in the tested samples. Regarding charge storage in MnO_2_ electrodes, two mechanisms have been suggested, as described in Eqs 8 and 9 [27–28]:

$${\text{MnO}}_{\text{2}}{\text{+Na}}^{\text{+}}\left({\text{or H}}^{\text{+}}\right){\text{ +e}}^{-}↔\text{MnOONa }\left(\text{or MnOOH}\right)$$ (8)

$${\text{NiO+OH}}^{-}↔{\text{NiOOH+e}}^{-}$$ (9)

Based on the reaction above, the OH− ion plays an important role in NiO charge storage. Indeed, decreasing the OH^−^ concentration in the Na_2_SO_4_ aqueous electrolyte solution might lead to less-significant contributions from NiO redox reactions; NiO mainly enhances the capacitance that corresponds to EDL storage [21,18]. The largest encircled area was exhibited by the CY7 electrode, indicating that this material has a higher electrochemical activity and capacitance. The calculated specific capacitance values for the CP, CA, and CY7 electrodes at a scan rate of 1 mVs^−1^ were 435, 458, and 500 Fg^−1^, respectively. The high specific capacitance of the CY7 electrode may be attributable to its lower agglomeration, well-distributed mesoporous structure, and low crystallinity (amorphousness), as discussed in the XRD, FESEM, and TEM sections. Therefore, the phase structure and morphology, in addition to the porous nanostructure, of MnO-NiO significantly influence its electrochemical properties.

**Fig 5:**
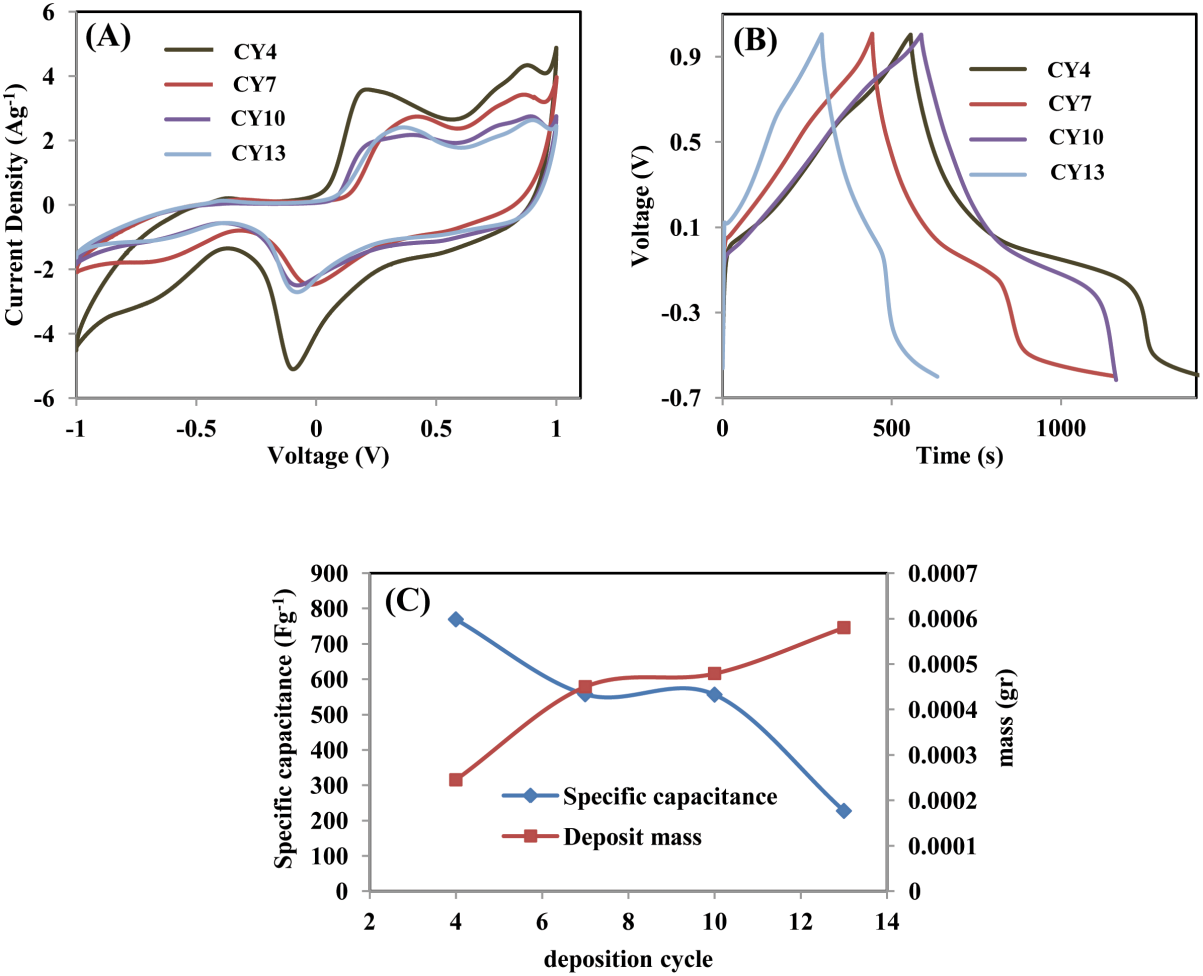
(A) CV curves at a scan rate of 5 mVs^−1^, (B) CD curve at a current density of 1 Ag^−1^ in a voltage range of −0.6 V to 1 V, and (C) specific capacitance determined from the discharge graph of the deposited mass versus the number of deposition cycles.

**Fig 6:**
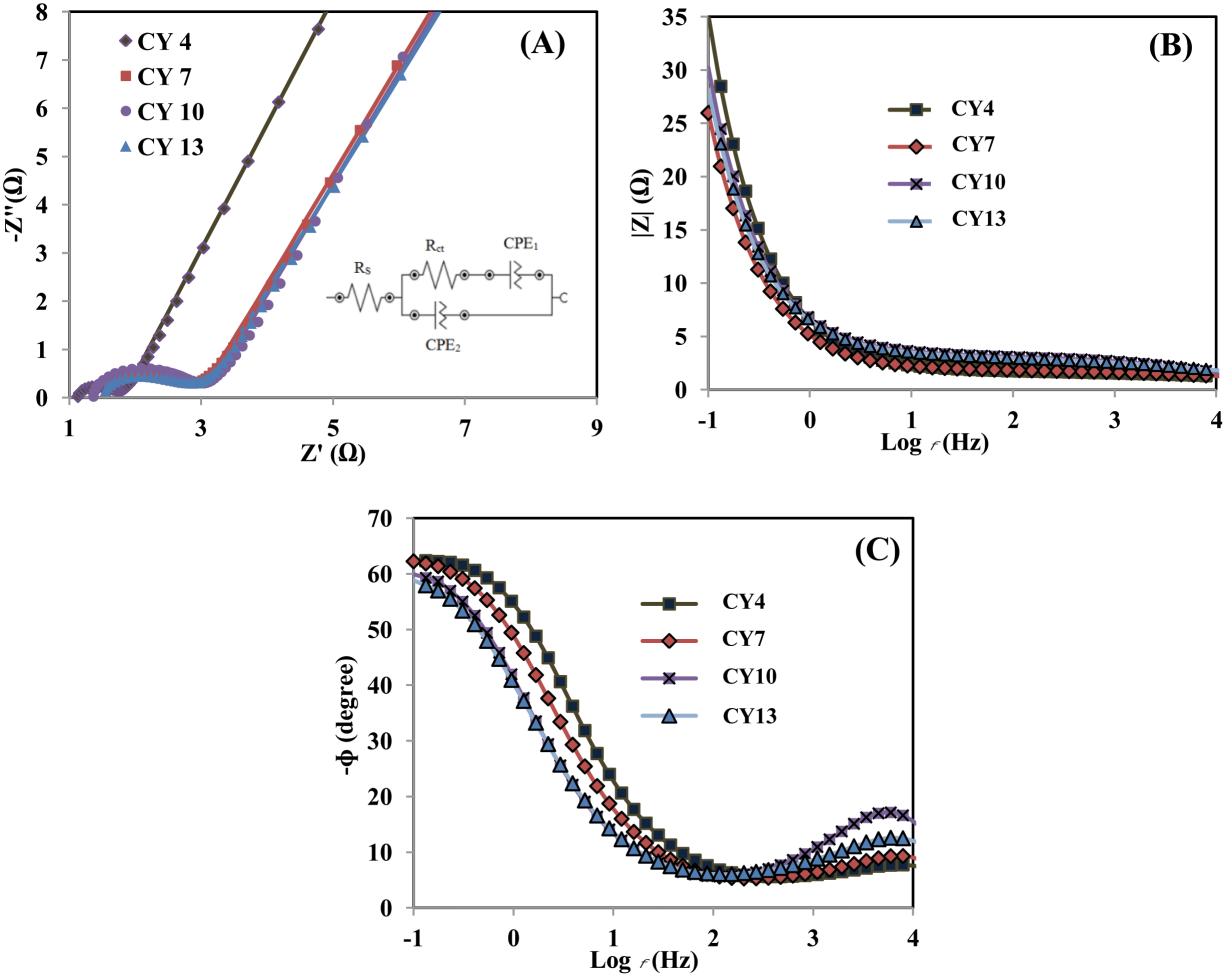
(A) Nyquist plot of all electrodes in the frequency range from 10 mHz to 100 kHz in Na_2_SO_4_ electrolyte, (B) Bode plots of the frequency’s dependence on the impedance magnitude (|Z|), (C) Bode plots of the frequency’s dependence on the phase angle (ϕ), and (D) specific capacitance retention until the 1000^th^ cycle.

To obtain additional information on the electrochemical behaviour of the synthesized CY7 electrode obtained using different numbers of deposition cycles, the CV curves of the CY4, CY7, CY10, and CY13 electrodes in 0.5-M Na_2_SO_4_ electrolyte were collected and are shown in Fig 5A. The peak current decreased as the number of deposition cycles increased, suggesting that the diffusion of cations into the MnO_2_-NiO electrode decreased. The denser, more compact flake structure may have prevented cations from migrating into the electrode material, thereby decreasing Na^+^ or OH^−^ ion adsorption. Fig 5B shows the CD curve of all electrodes obtained with different numbers of deposition cycles at a current density of 1 Ag^−1^ and a voltage range from −0.6 V to 1 V. The non-linearity of the curve is attributable to the faradic redox reaction that occurs in this voltage range [14,29]. The CY4 electrode exhibited a longer discharge time, indicating that its charge-storage behaviour is more efficient than those of other electrodes. The specific capacitances of CY4, CY7, CY10, and CY13, based on the mass of deposited MnO-NiO calculated from a CDC curve, were 769 Fg^−1^, 557 Fg^−1^, 556 Fg^−1^, and 227 Fg^−1^, respectively. The trend of the specific capacitance was inversely proportional to the mass of the electrode [30], as shown in Fig 5C. The observed decreasing trend of the capacitance relative to the mass is attributable to the increasing resistance, which influences the ion conduction, as shown in the Nyquist plot in Fig 6.

The ion transport resistances of all samples was investigated by EIS. The equivalent circuit based on the Nyquist plots (Fig 6A) was fitted using Nova software, and the parameters are shown in Table 1. R_s_ was determined from the x-intercept of the arc in the high-frequency region, which reflects the ohmic resistance of the electrolyte, the contact resistance, and the internal resistance of the material. Because R_ct_ was measured in the semicircle region, it corresponds to the transfer resistance of the CD reaction at the electrolyte/electrode interface [31]. The R_s_ and R_ct_ values of the CY4 electrode were smaller than those of the CY7, CY10, and CY13 electrodes, as revealed in Table 1 (Fig 6A). This finding indicates good wettability of the electrode/electrolyte interfaces and suggests that the cation intercalation/deintercalation activity into or from the CY4 electrode is easier than those of the other electrodes [14]. Additionally, the CY4 electrode’s smaller R_s_ values indicate that it has lower resistivity, which may be the result of the increased surface area of the deposited composite electrode structure [32]. Thus, the CY4 electrode has higher specific capacitance and exhibits better electrochemical performance than the CY7, CY10, and CY13 electrodes.

**Table 1:** Equivalent circuit parameters deduced by fitting Nyquist plots and the frequencies at ɸ = −45° for all electrodes.

Number of deposition cycles	R_s_ (Ω)	R_ct_ (Ω)	*f*_ɸ = ‒45°_ (Hz)
4	1.10	0.75	2.22
7	1.29	1.78	1.26
10	1.30	1.8	0.72
13	1.31	1.8	0.63

To obtain additional more information on the processes that contribute to the electrode’s capacitance, the Bode plots presented in Fig 6B and 6C can be divided into three frequency regions: high- (*f*>10 Hz), medium-, and low-frequency regions (*f*<1 Hz). Typical capacitive behaviour was observed in both plots throughout the whole frequency range. In the low-frequency region, all deposited films presented a slope of ~−1 in the *f*-|Z| plots. The phase angles in the *f*-ɸ plots were between −70° and −55°. The intermediate values of the impedance magnitude ǀZǀ and phase angle (ɸ) were obtained in the medium-frequency region, whereas in the high-frequency region, the capacitance was nearly zero because the |Z| is independent. Additionally, the phase angle began to decrease to almost zero as the frequency was further increased. The frequency at a phase angle = −45° in the *f*-ɸ plots (Fig 6C) is known as the frequency response region, and higher frequencies at ɸ = −45° indicate better capacitive responses. The highest frequency response found here was 2.22 Hz for the CY4 electrode, and this value was related to this material’s reasonably fast electron/proton transport response, which led to high capacitance [33].

**Fig 7:**
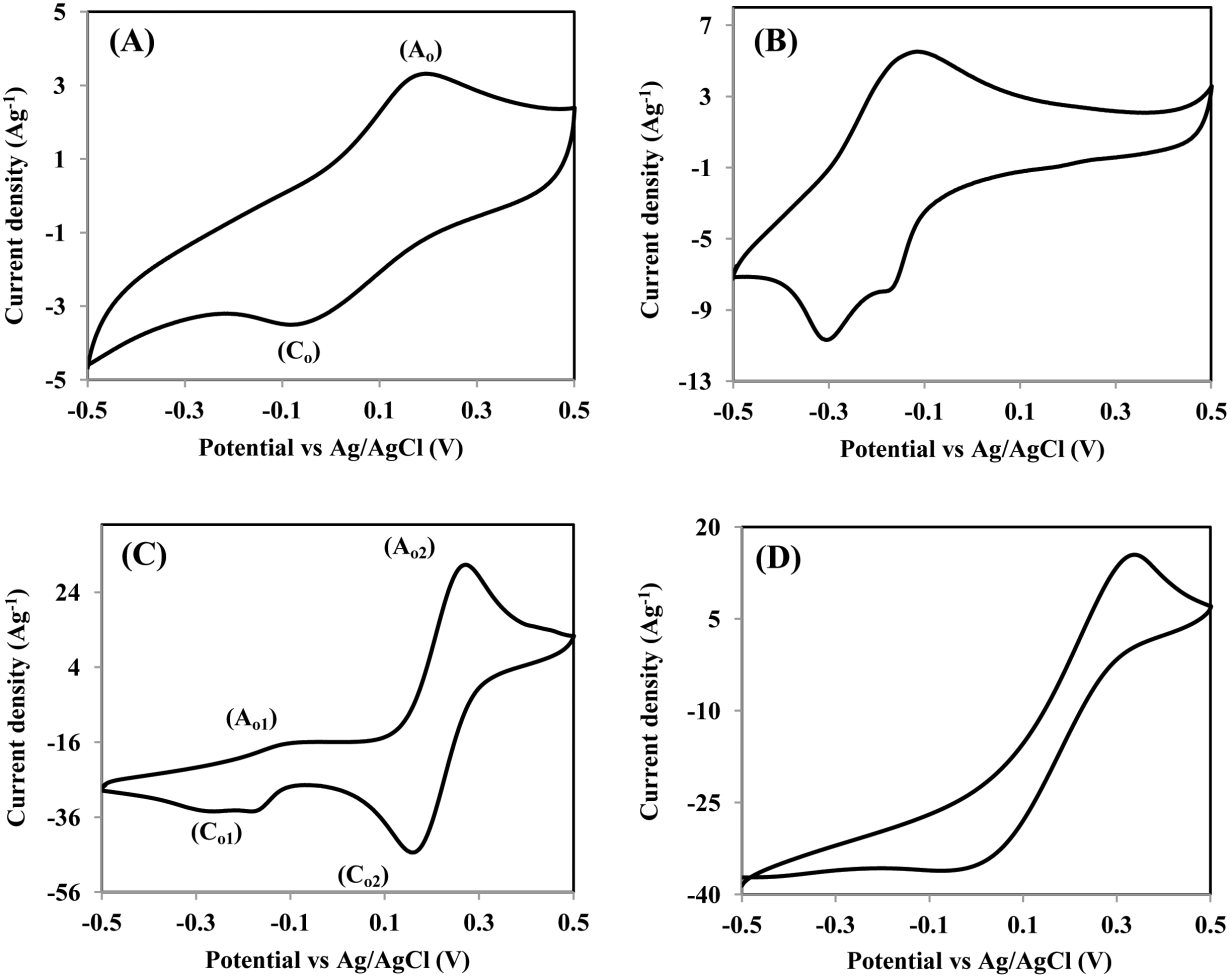
CV curves of CY4 at a scan rate of 5 mVs^−1^ within the potential range of −0.5 V to 0.5 V in (A) 0.5-M Na_2_SO_4_, (B) 0.5-M KOH, (C) mixed 0.5-M KOH/0.04-M K_3_Fe(CN)_6_, and (D) 0.04-M K_3_Fe(CN)_6_.

After confirming that the CY4 electrode was the optimum electrode in this system, its performance was further investigated in different alkaline electrolytes. The CVs of the CY4 electrode in 0.5-M Na_2_SO_4_, 0.5-M KOH, 0.04-M K_3_Fe(CN)_6_, and mixed 0.5-M KOH/0.04-M K_3_Fe(CN)_6_ electrolytes were obtained sweeping the potential from −0.5 V to 0.5 V at a scan rate of 5 mVs^−1^ and are shown in Fig 7. A well-defined pair of anodic peaks (A_0_) and a cathodic peak (C_0_) are observed cantered at potentials of approximately +0.20 and −0.05 V (vs. Ag/AgCl), respectively, in Na_2_SO_4_ electrolyte (Fig 7A). These peaks can be attributed to the redox reaction dominated by MnO_2_, according to Eqs 8 and 9 (presented above). The KOH electrolyte was chosen because it has a higher OH^−^ concentration, which plays an important role in NiO reactions during charging/discharging (Eq 9) [34]. The combined contributions of MnO_2_-NiO in the KOH electrolyte increased the current response, as shown in Fig 7B. The high current response of this system results from the complete utilization of NiO and the relatively small K^+^ cation radius (3.31 Å) compared to that of Na^+^ ions (3.35 Å), which facilitates the passage of K^+^ ions into the electrode matrix during the charging process [35].

When 0.04-M K_3_Fe(CN)_6_ was added to the 0.5-M KOH electrolyte, an additional pair of anodic peaks at approximately +0.25 V (A_02_) and a cathodic peak at +0.17 V (C_02_) arose (Fig 7C). These peaks were attributed to the redox reaction of K_4_Fe(CN)_6_ to K_3_Fe(CN)_6_, which is consistent with the CV plot obtained using K_3_Fe(CN)_6_ electrolyte (Fig 7D) [36]. The drastic increase in the current response observed in this system occurred because of the appearance of two significant faradic reactions originating from the redox couple [Fe(CN)_6_]^3−^/[Fe(CN)_6_]^4−^ in the electrolyte (Eq 10) and the redox reactions in the highly electroactive electrodes (Eq 11).

$$\text{Redox electrolyte: }{\left[\text{Fe}{\left(\text{CN}\right)}_{\text{6}}\right]}^{3-}{\text{+ e}}^{-}↔\;{\left[\text{Fe}{\left(\text{CN}\right)}_{\text{6}}\right]}^{4-}$$ (10)

$${\text{Redox electrode: M}}^{\text{z+}}↔{\text{ M}}^{\left(\text{z+n}\right)\text{+}}{\text{+ ne}}^{-}$$ (11)

where M is the Ni^2+^ or Mn^2+^ cation, and 1≤n≤z.

**Fig 8:**
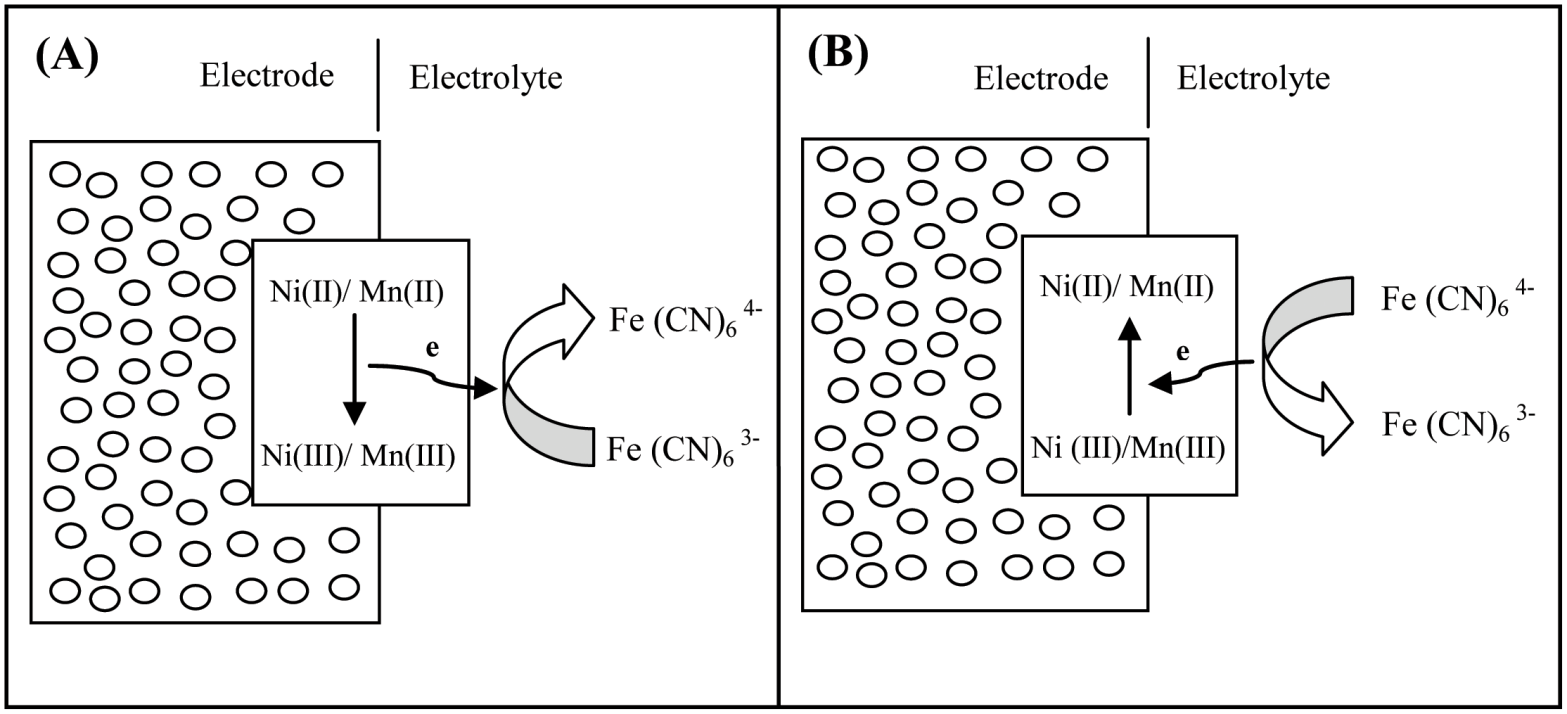
Schematic of the role of hexacyanoferrate (II) and (III) in the processes of (A) charge and (B) discharge in the CY4 electrode.

The detailed mechanism was presented in a previous study [37]. When the electrode is charged, the active materials are oxidized, and the electrolyte [Fe(CN)_6_]^3−^ accepts an electron via the reduction of hexacyanoferrate (III) to (II); the hexacyanoferrate ions act as “electron carriers” (Fig 8A). When the electrode is discharged, the reaction is reversed: The hexacyanoferrate ions act as “electron donors”, which means that [Fe(CN)_6_]^4−^ is converted to [Fe(CN)_6_]^3−^, and the released electrons reduce the electrode active materials (Fig 8B). This helps to improve the capacitive performance of the active material [37–38]. The specific capacitances calculated from the CV at 5 mVs^−1^ are as follows: 474 Fg^−1^, 780 Fg^−1^, and 5130 Fg^−1^ for Na_2_SO_4_, KOH, and mixed KOH/K_3_Fe(CN)_6_, respectively. The importance of K_3_Fe(CN)_6_ was confirmed by the enhancement of the specific capacitance.

**Fig 9:**
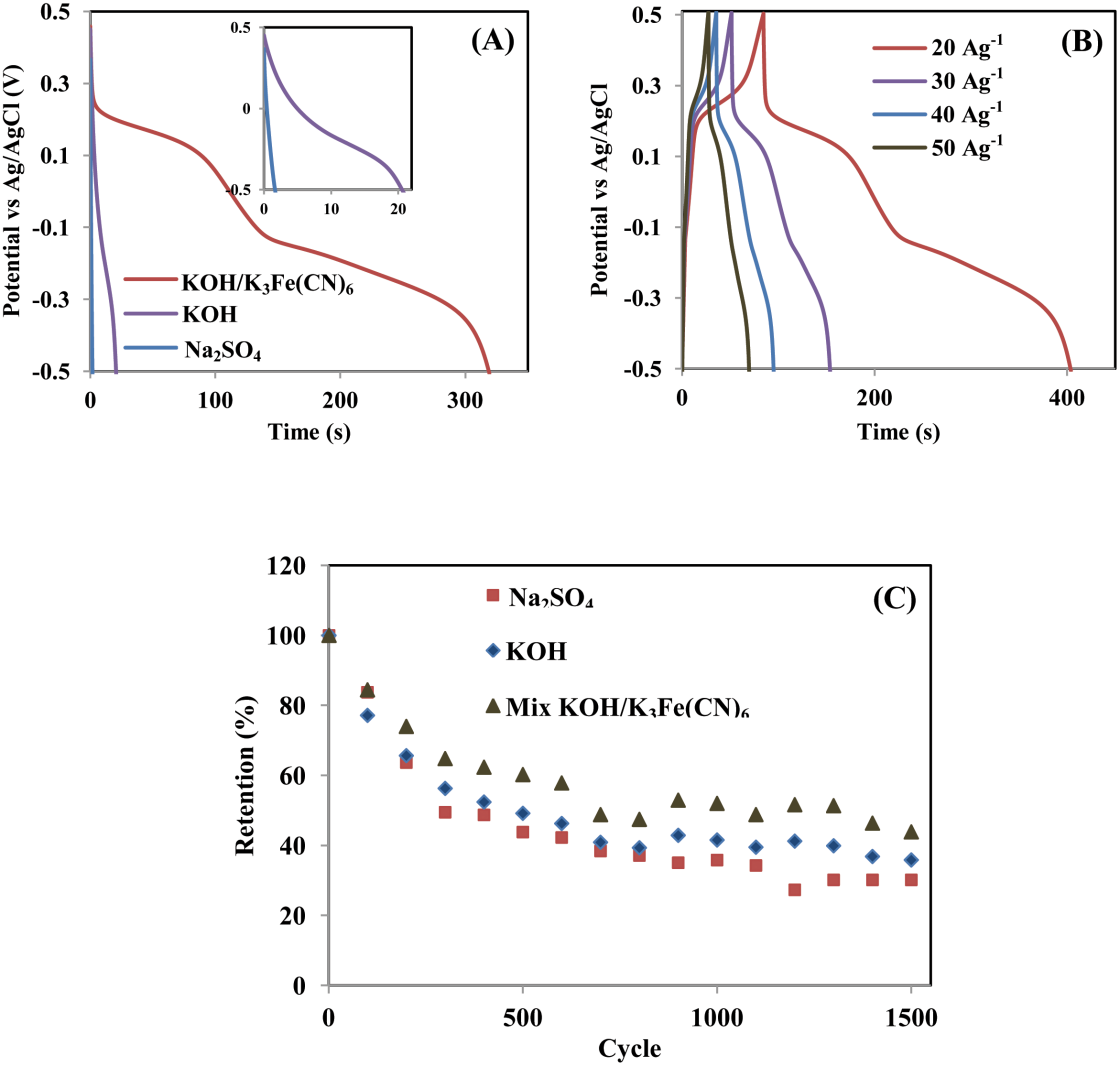
(A) CD profiles of the CY4 electrode’s current density at 20 Ag^−1^ in three different electrolytes, (B) CD profiles collected using different applied current densities in mixed KOH/K_3_Fe(CN)_6_, and (C) specific capacitance retention after the 1500^th^ cycle at a scan rate of 10 mVs^−1^ in the potential range from −0.5 V to 0.5 V.

The CD profiles of the CY4 electrode in Na_2_SO_4_, KOH, and mixed KOH/ K_3_Fe(CN)_6_ electrolytes at a current density of 20 Ag^−1^ are shown in Fig 9A. The specific capacitances calculated from the discharge curves were 46 Fg^−1^, 583 Fg^−1^, and 3509 Fg^−1^ in Na_2_SO_4_, KOH, and mixed KOH/K_3_Fe(CN)_6_, respectively. A high specific capacitance was also obtained in mixed KOH/K_3_Fe(CN)_6,_ which is consistent with the CV results. The CD of CY4 in mixed KOH/K_3_Fe(CN)_6_ electrolyte was further investigated using different applied current densities and is plotted in Fig 9B. The obviously non-linear curve, which includes two potential plateaus at approximately −0.14 V and 0.18 V, was inferred to result from the redox reaction of KOH and K_3_Fe(CN)_6_ [29]. The energy and power densities of CY4 in mixed KOH/ K_3_Fe(CN)_6_ were 1322 Wh kg^−1^ and 110.5 kW kg^−1^, respectively, at a current density of 20 Ag^−1^.

The cycling performance of the CY4 electrode in three different electrolytes is shown in Fig 9D. The capacitance retention exhibited a drastic decrease during continuous charging-discharging. The specific capacitance retention values in Na_2_SO_4_, KOH, and mixed KOH/K_3_Fe(CN)_6_ electrolytes after 1500 cycles were 30%, 36%, and 44%, respectively. The addition of 0.4-M K_3_Fe(CN)_6_ to the KOH electrolyte resulted in a small improvement in the electrode stability. The high loss of specific capacitance in this system could be attributed to substantial electrode degradation resulting from the high current passed through it during the cyclability test and volume loss of the active material [38,39]. Although the specific capacitance and energy density of this system were extremely high, its stability must be improved.

## Conclusions

We have fabricated MnO_2_-NiO composite electrodes with a network-like structure using three electrodeposition modes: CP, CA, and CV. The composite CY7 electrode exhibited high homogeneity, a highly amorphous nature, and good capacitive behaviors compared to the CA and CP electrodes. The specific capacitances based on the mass of deposited MnO_2_-NiO in the CP, CA, and CY7 electrode were 435 Fg^−1^, 458 Fg^−1^, and 500 Fg^−1^, respectively, and thus, CV electrodeposition was identified as the most suitable mode for MnO_2_-NiO composite electrode electrodeposition. The number of deposition cycles was also found to control the nucleation process, morphology, deposit thickness, and electrochemical performance of the resulting electrodes. Indeed, the capacitance decreased as the number of deposition cycles increased because of the weak wettability properties at the electrode/electrolyte interfaces and insufficient intercalation/deintercalation activity into or from the electrode. The best-performing electrode was found to be the CY4 electrode based on a comparison performed in Na_2_SO_4_ electrolyte. The optimum electrochemical performance of the CY4 electrode was achieved in mixed KOH/K_3_Fe(CN)_6_ electrolyte, resulting in a specific capacitance of 3509 Fg^−1^ and energy and power densities of 1322 Wh kg^−1^ and 110.5 kW kg^−1^, respectively, at a current density of 20 Ag^−1^. The simple electrodeposition of MnO_2_-NiO composite electrodes is cost efficient, versatile, and scalable for the synthesis of composite electrodes for supercapacitor applications.

## References

[1] Suhasini. Effect of deposition method and the surfactant on high capacitance of electrochemically deposited MnO2 on stainless steel substrate. J Electroanal Chem. 2013;690: 13–18.

[2] Wei WF, Cui XW, Chen WX, Ivey DG. Manganese oxide-based materials as electrochemical supercapacitor electrodes. Chem Soc Rev. 2011;40: 1697–1721. 10.1039/c0cs00127a 21173973

[3] Conway BE. Electrochemical supercapacitors: Scientific fundamentals and technological applications. New York: Kluwer-Plenum; 1999.

[4] Li Y, Xie H, Wang J, Chen L. Preparation and electrochemical performances of α-MnO_2_ nanorod for supercapacitor. Mater Lett. 2011;65: 403–405.

[5] Babakhani B, Ivey DG. Anodic deposition of manganese oxide electrodes with rod-like structures for application as electrochemical capacitors. J Power Sources. 2010;195: 2110–2117.

[6] Zhang Y, Feng H, Wu X, Wang L, Zhang A, Xia T, et al Progress of electrochemical capacitor electrode materials: A review. Int J Hydrogen Energ. 2009;34: 4889–4899.

[7] Lokhande CD, Dubal DP, Joo OS. Metal oxide thin film based supercapacitors. Curr Appl Phys. 2011;11: 255–270.

[8] Khoh WH, Hong JD. Solid-state asymmetric supercapacitor based on manganese dioxide/reduced-graphene oxide and polypyrrole/reduced-graphene oxide in a gel electrolyte. Colloids Surf, A. 2014;456: 26–34.

[9] Prasad KPS, Dhawale DS, Joseph S, Anand C, Wahab MA, Mano A, et al Post-synthetic functionalization of mesoporous carbon electrodes with copper oxide nanoparticles for supercapacitor application. Micropor Mesopor Mat. 2013;172: 77–86.

[10] Wang H, Gao Q, Hu J. Asymmetric capacitor based on superior porous Ni–Zn–Co oxide/hydroxide and carbon electrodes. J Power Sources, 2010;195: 3017–3024.

[11] Sun K, Wang H, Peng H, Wu Y, Ma G, Lei Z. Manganese oxide nanorods supported on orange peel-based carbon nanosheets for high performance supercapacitors. Int J Electrochem Sci. 2015;10: 2000–2013.

[12] Kim SI, Lee JS, Ahn HJ, Song HK, Jang JH. Facile route to an efficient NiO supercapacitor with a three dimensional nanonetwork morphology. ACS Appl Mater Interfaces. 2013;5: 1596–1603. 10.1021/am3021894 23373659

[13] Vijayakumar S, Nagamuthu S, Muralidharan G. Porous NiO/C nanocomposites as electrode material for electrochemicalsupercapacitors. ACS Sustainable Chem Eng. 2013;1: 1110–1118.

[14] Rusi, Majid SR. High performance super-capacitive behaviour of deposited manganese oxide/nickel oxide binary electrode system. Electrochim Acta. 2014;138: 1–8.

[15] Sorkhabi HA, Asghari E, La'le Badakhshan P. Potentiostatic and cyclic voltammetric deposition of nanostructured manganese oxide for supercapacitor applications. Curr Appl Phys. 2014;14: 187–191.

[16] Jagadale AD, Kumbhar VS, Lokhande CD. Supercapacitive activities of potentiodynamically deposited nanoflakes of cobalt oxide (Co3O4) thin film electrode. J Colloid Interface Sci. 2013;406: 225–230. 10.1016/j.jcis.2013.05.037 23827481

[17] Dubal DP, Dhawale DS, Gujar TP, Lokhande CD. Effect of different modes of electrodeposition on supercapacitive properties of MnO_2_ thin films. Appl Surf Sci. 2011;257: 3378–3382.

[18] Rusi, Majid SR. Electrodeposited Mn_3_O_4_-NiO-Co_3_O_4_ as a composite electrode material for electrochemical capacitor. Electrochim Acta. 2014;175: 193–201.

[19] Wang G, Zhang L, Zhang J. A review of electrode materials for electrochemical supercapacitors. Chem Soc Rev. 2012;41(2): 797–828. 10.1039/c1cs15060j 21779609

[20] Zhang Y, Yang Y, Zhang Y, Zhang T, Ye M. Heterogeneous oxidation of naproxen in the presence of α-MnO_2_ nanostructures with different morphologies. Appl Catal, B. 2012;127: 182–189.

[21] Wu MS, Huang YA, Yang CH, Jow JJ. Electrodeposition of nanoporous nickel oxide film for electrochemical capacitors. Int J Hydrogen Energ. 2007;32: 4153–4159.

[22] Yousefi T, Golikand AN, Mashhadizadeh MH, Aghazadeh M. Template-free synthesis of MnO_2_ nanowires with secondary flower like structure: Characterization and supercapacitor behavior studies. Curr Appl Phys. 2012;12: 193–198.

[23] Hwang BJ, Santhanam R, Lin Y. Nucleation and growth mechanism of electropolymerization of polypyrrole on gold/highly oriented pyrolytic graphite electrode. J Electrochem Soc. 2000;147: 2252–2257.

[24] Babakhani B, Ivey DG. Effect of electrodeposition conditions on the electrochemical capacitive behavior of synthesized manganese oxide electrodes. J Power Sources. 2011;196: 10762–10774.

[25] Liu Y, Zhang M, Zhang J, Qian Y. A simple method of fabricating large-area α-MnO_2_ nanowires and nanorods. J Solid State Chem. 2006;179: 1757–1761.

[26] Li H, Zhu S, Xi H, Wang R. Nickel oxide nanocrystallites within the wall of ordered mesoporous carbon CMK-3: Synthesis and characterization. Micropor Mesopor Mat. 2006;89: 196–203.

[27] Cross A, Morel A, Cormie A, Hollenkamp T, Donne S. Enhanced manganese dioxide supercapacitor electrodes produced by electrodeposition. J Power Sources. 2011;196: 7847–7853.

[28] Li SH, Liu QH, Qi L, Lu LH, Wang HY. Progress in research on manganese dioxide electrode materials for electrochemical capacitors. Chinese J Anal Chem. 2012;40: 339–346.

[29] Dubal DP, Gund GS, Holze R, Jadhav HS, Lokhande CD, Park CJ. Solution-based binder-free synthetic approach of RuO_2_ thin films for all solid state supercapacitors. Electrochim Acta. 2013;103: 103–109.

[30] Broughton JN, Brett MJ. Variations in MnO_2_ electrodeposition for electrochemical capacitors. Electrochim Acta. 2005;50: 4814–4819.

[31] Chen Y, Wang JW, Shi XC, Chen BZ. Pseudocapacitive characteristics of manganese oxide anodized from manganese coating electrodeposited from aqueous solution. Electrochim Acta. 2013;109: 678–683.

[32] Hughes M, Chen GZ, Shaffer MSP, Fray DJ, Windle AH. Electrochemical capacitance of a nanoporous composite of carbon nanotubes and polypyrrole. Chem Mater. 2002;14: 1610–1613.

[33] Ding R, Qi L, Jia M, Wang H. Facile and large-scale chemical synthesis of highly porous secondary submicron/micron-sized NiCo_2_O_4_ materials for high-performance aqueous hybrid AC-NiCo_2_O_4_ electrochemical capacitors. Electrochim Acta. 2013;107: 494–502.

[34] Wu MS, Huang YA, Yang CH, Jow JJ. Electrodeposition of nanoporous nickel oxide film for electrochemical capacitors. Int J Hydrogen Energ. 2007;32(17): 4153–4159.

[35] Nithya VD, Kalai Selvan R, Kalpana D, Vasylechko L, Sanjeeviraja C. Synthesis of Bi_2_WO_6_ nanoparticles and its electrochemical properties in different electrolytes for pseudocapacitor electrodes. Electrochim Acta. 2013;109: 720–731.

[36] Zhao C, Zheng W, Wang X, Zhang H, Cui X, Wang H. Ultrahigh capacitive performance from both Co(OH)_2_/graphene electrode and K_3_Fe(CN)_6_ electrolyte. Sci Rep. 2013;3: 2986 10.1038/srep02986 24136136 PMC3798881

[37] Chen K, Song S, Xue D. An ionic aqueous pseudocapacitor system: electroactive ions in both a salt electrode and redox electrolyte. RSC Adv. 2014;4(44): 23338–23343.

[38] Rusi, Chan PY, Majid SR. Layer by layer ex-situ deposited cobalt-manganese oxide as composite electrode material for electrochemical capacitor. PLoS ONE 2015;10(7): e0129780 10.1371/journal.pone.0129780 26158447 PMC4497717

[39] Engstrom AM, Doyle FM. Exploring the cycle behavior of electrodeposited vanadium oxide electrochemical capacitor electrodes in various aqueous environments. J Power Sources. 2013;228: 120–131.

